# Diagnostic accuracy of upper gastrointestinal series in children with suspected intestinal malrotation

**DOI:** 10.1007/s13304-023-01559-8

**Published:** 2023-06-16

**Authors:** Mattioli Girolamo, Gallo Emanuela, Wong Michela Cing Yu, Marzoli Anna, Pongiglione Marta, Calevo Maria Grazia, Paolo Gandullia, Serena Arrigo, Avanzini Stefano, Damasio Maria Beatrice

**Affiliations:** 1 University of Genoa, DINOGMI, Genoa, Italy; 2 Pediatric Surgery Unit, IRCCS, Istituto Giannina Gaslini, Via G. Gaslini 5, 16147 Genoa, Italy; 3 Radiology Unit, IRCCS, Istituto Giannina Gaslini, Via G. Gaslini 5, 16147 Genoa, Italy; 4 Epidemiology and Biostatistics Unit, Scientific Direction, IRCCS Istituto Giannina Gaslini, Via G. Gaslini 5, 16147 Genoa, Italy; 5 Pediatric Gastroenterology and Endoscopy Department, IRCCS, Istituto Giannina Gaslini, 16147 Genoa, Italy; 6 Via Gerolamo Gaslini, 5, 16148 Genoa, Italy

**Keywords:** Duodenal–Jejunal Junction, Treitz ligament, Midgut volvulus, Recurrent vomiting, Intestinal Malrotation Diagnosis

## Abstract

Intestinal malrotation (IM) results from an altered or incomplete rotation of the fetal midgut around the superior mesenteric artery axis. The abnormal anatomy of IM is associated with risk of acute midgut volvulus which can lead to catastrophic clinical consequences. The upper gastro-intestinal series (UGI) is addressed as the gold standard diagnosis procedure, but a variable failure degree has been described in literature. The aim of the study was to analyze the UGI exam and describe which features are the most reproducible and reliable in diagnosing IM. Medical records of patients surgically treated for suspected IM between 2007 and 2020 at a single pediatric tertiary care center were retrospectively reviewed. UGI inter-observer agreement and diagnostic accuracy were statistically calculated. Images obtained with antero-posterior (AP) projections were the most significant in terms of IM diagnosis. Duodenal-Jejunal Junction (DJJ) abnormal position resulted to be the most reliable parameter (Se = 0.88; Sp = 0.54) as well as the most readable, with an inter-reader agreement of 83% (*k* = 0.70, CI 0.49–0.90). The First Jejunal Loops (FJL), caecum altered position and duodenal dilatation could be considered additional data. Lateral projections demonstrated an overall low sensitivity (Se = 0.80) and specificity (Sp = 0.33) with a PPV of 0.85 and a NPV of 0.25. UGI on the sole AP projections ensures a good diagnostic accuracy. The position of the third portion of the duodenum on lateral views showed an overall low reliability, therefore it was not helpful but rather deceiving in diagnosing IM.

## Introduction

Intestinal Malrotation (IM) is a potentially life-threatening congenital condition whose diagnosis is often challenging. IM is determined by an abnormal gut morphogenesis, therefore an alteration of the sequence of herniation, counterclockwise rotation around the superior mesenteric artery (SMA) axis [1] and fixation of the fetal midgut. If the cecocolic loop returns to the abdomen before the midgut a nonrotation may result, this condition is generally associated with a wide based mesentery and, therefore, is not at risk of midgut volvulus. Classic IM presentation derives from an aberrant rotation of the fetal midgut presenting itself with the duodenojejunal junction (DJJ) fixed to the right upper quadrant, the presence of adhesive bends which can attach the gallbladder and the cecum to the abdominal wall and above all a narrow mesenteric base which predisposes to potentially fatal midgut volvulus. Another presentation may be the reverse rotation resulting from a clockwise 90° rotation in which the duodenum lies anteriorly to the SMA and the colon which may lead to partial mesenteric obstruction or hernias [1-4].

All these types of IM may present with various degree of anatomical dysmorphism and thus affecting differently the clinical onset [1, 2]. As a matter of fact, IM can present itself either in the early life or afterwards and either as surgical emergency with a midgut volvulus or with various digestive chronic symptoms [5, 6].

This clinical variability makes IM diagnosis often challenging justifying the need for a reliable and standardized diagnostic protocol which, to date, lacks. Particularly, in pediatric population IM remains a surgical diagnosis. Upper Gastrointestinal series (UGI), the most frequent used diagnostic procedure, has been associated with a variable failure degree [7-10]. Plus, the use of US-Color Doppler to detect the inversion of relative position of the SMA and the superior mesenteric vein (SMV) and possibly the presence of a whirlpool sign (SMV wrapping clock wisely around the SMA in case of volvolus) still remains operator-dependent [9, 11].

Facing the IM’s potentially catastrophic clinical consequences, especially in the first year of life, and the lack of a strong diagnostic tool surgeons and radiologists alike need to take under consideration the diagnostic performance and the reliability of UGI [8, 9, 12].

A review of UGI studies from patients who underwent surgery because of suspected IM was performed with the aim to determine which features can lead to a prompt and consistent diagnosis and which, instead, risk to jeopardize it.

## Methods

A retrospective review was conducted based on medical records of patients surgically treated for IM between January 2007 and December 2020 at a single pediatric tertiary care center. The present study was conducted with the approval of the Regional Ethical Review Board (321/2022–DB id 12,450).

Inclusion criteria covered surgical treatment for suspected IM and a complete pre-operative UGI imaging report. All patients who were affected by syndromic conditions associated with malrotation such as gastroschisis, omphalocele, congenital diaphragmatic hernia or situs inversus, and those who had not been studied with UGI, or those who had a previous abdominal surgery were excluded. Medical records of patients admitted with suspected IM and which subsequently underwent abdominal surgery in the study period were reviewed in order to identify potentially eligible patients.

Collected data included sociodemographic data (age and sex), clinical variables (symptoms and onset presentation), imaging findings and operative records.

Medical history and imaging findings were analyzed separately by two radiologists blinded to patients groups. Five following different UGI aspects were reviewed: (1) duodenal-jejunal junction (DJJ) position; (2) the orientation of the third portion of the duodenum on lateral views; (3) presence of duodenal dilatation; (4) position of first jejunal loops (FJL); (5) position of the caecum.

**Fig. 1:**
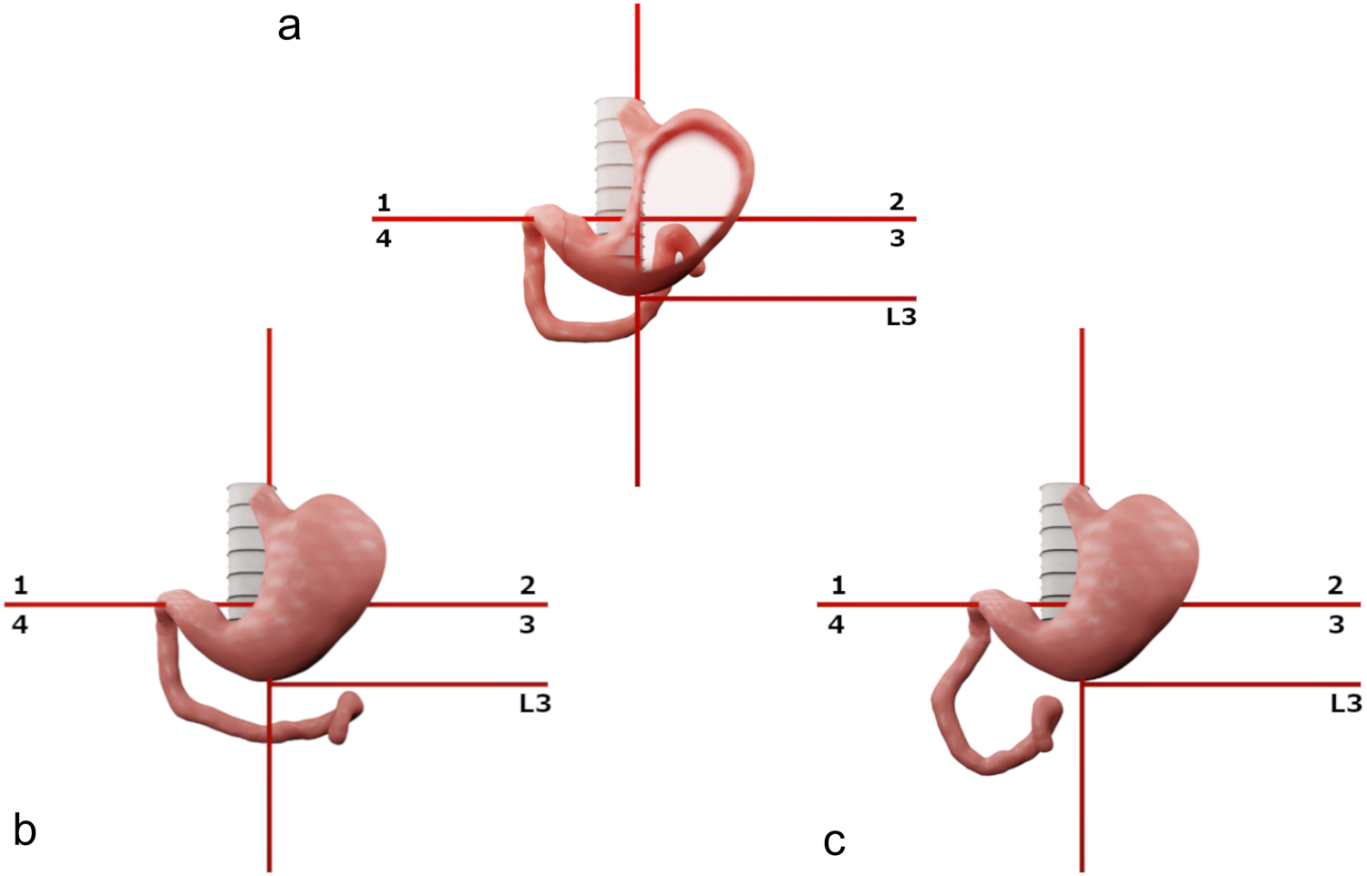
Graphic tool used to objectivate the position of anatomic structures on dynamic Upper Gastro-Intestinal scans. Figure shows the grid used in the study to objectivate the position of the duodenal-Jejunal Junction (DJJ) when superimposed on Upper Gastro-Intestinal seires (UGI) scans. The grid is designed with two imaginary lines crossing at the left of the spine at the level of the pylorus and a third line positioned at the level of the end-plate of the third lumbar vertebra. **a** normal DJJ position; **b** low positioned DJJ may be a sign of intestinal malrotation; **c** most frequent position of DJJ in malrotated patients

As described in literature, DJJ was considered normal whenever depicted at the left of the spine approximately at the level of the pylorus, not below the level of the vertebral body of L2-L3 [13-15]. In this study, a grid of two imaginary lines was subsequently elaborated to better visualize the DJJ on AP projections and to facilitate the reproducibility of the lecture. Superimposing the grid on UGI radiographs would help in locating the DJJ in four quadrants as previously described by Friederick et al. [13] Furthermore, the analysis of Katz et al. over the normal variation of DJJ position in children guided the process of introducing an additional horizontal line dividing the third quadrant into two halves at the level of superior end-plate of the 3rd lumbar vertebra (L3 quadrant, Fig. 1) [14]. Hence, in this study the position of DJJ was evaluated and then considered within the grid to collect data. When low placed the DJJ is considered significant for IM, in the grid presented the low placed DJJ falls into the L3 quadrant. On AP projections, the presence of dilatation of the second or the third duodenal portion, the position of the FJL, and eventually the caecum position were also reviewed and later considered within the grid to facilitate the reproducibility of the lecture. On lateral views the third portion of the duodenum was evaluated and considered normal when depicted retroperitoneally [7, 16, 17].

Whenever available, US Color-doppler to detect the superior mesenteric vessels relative position was reviewed as additional data and was not used in any case to validate UGI analysis.

Medical history and operative records were reviewed by a consultant surgeon blinded to the UGI records. Presence of abnormal congenital adhesions, namely Ladd’s bands, and the DJJ laying at the right of the spine were the two surgical criteria considered significant for IM. Cecal position was not considered intraoperative criteria of IM since it is generally fixed in place by Ladd’s bands [1]. Surgical diagnosis with these two criteria was considered the reference standard.

Statistical analyses were performed with SPSS (version 20.0, IBM, NY, USA).

Descriptive statistics were generated for the whole cohort and data were expressed as mean and standard deviation for continuous variables. Median value and range were calculated and reported, as were absolute or relative frequencies for categorical variables. Categorical data were compared between groups using the Chi-squared test or Fisher’s exact test. Interobserver agreement was defined using Cohen's kappa (*κ*).

Kappa values were interpreted using the following cut-offs: < 0 poor, 0.00–0.20 slight, 0.21–0.40 fair, 0.41–0.60 moderate, 0.61–0.80 substantial, 0.81–1 almost perfect [18]. Then, sensitivity (Se), specificity (Sp), positive predictive value (PPV), negative predictive value (NPV) were calculated. A *p*-values < 0.05 was considered statistically significant.

## Results

**Fig. 2:**
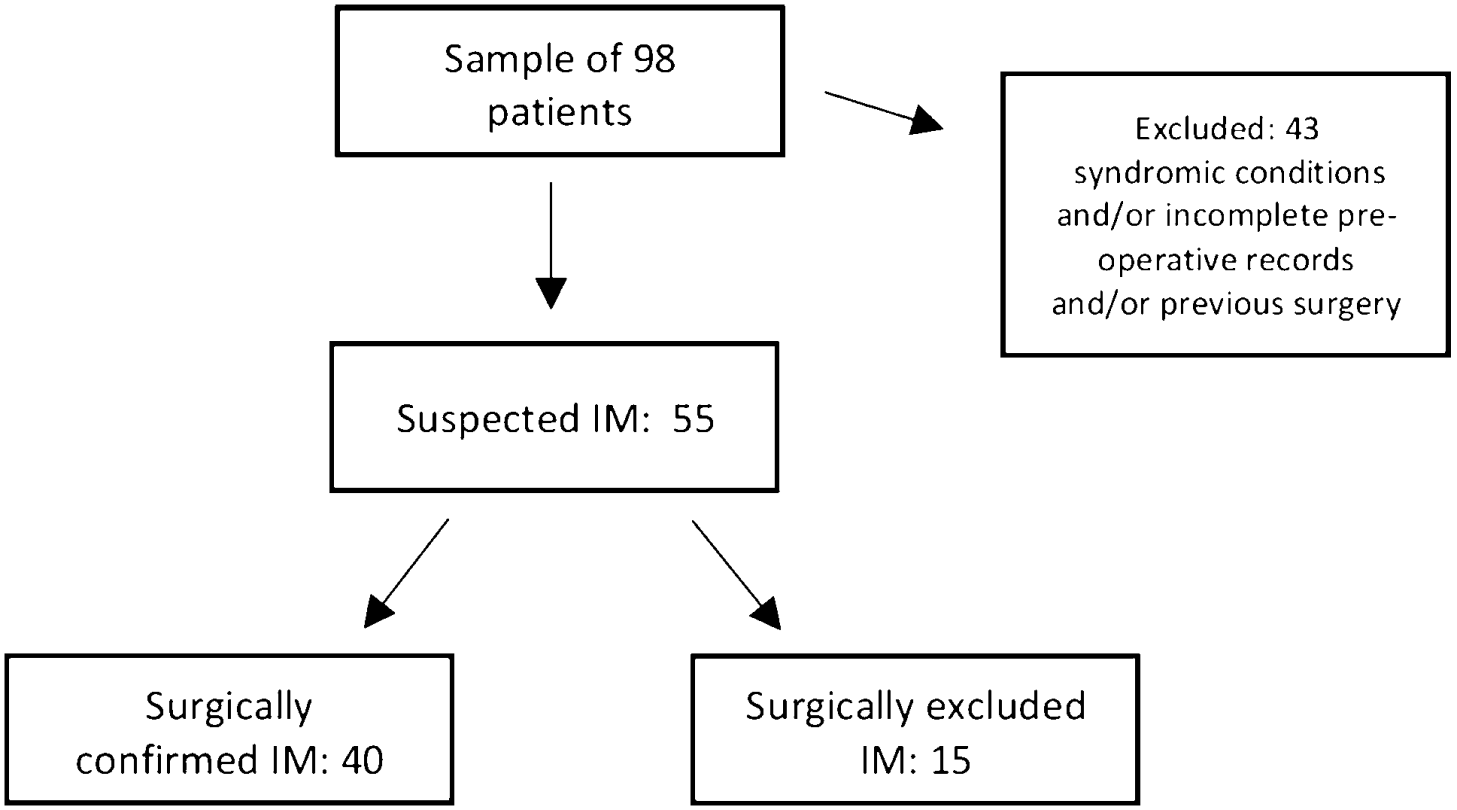
Diagram shows flow of participants of the study. The sample of consecutive patients admitted between 2007 and 2020 with suspected Intestinal Malrotation (IM) was made of 98 patients. Subsequently 43 patients were excluded due to inclusion criteria. Thus, the study population was made of 55 patients admitted because of suspected IM, 40 of them were surgically diagnosed with it, while 15 resulted non-malrotated at the surgical procedure

In the study period 98 consecutive patients had been surgically treated for suspected IM. Fifty-five patients fulfilled the inclusion criteria of the study and 40 (73%) were eventually confirmed with IM at the surgical operation. (Fig. 2) Distribution of the 43 patients excluded was analyzed: 29 patients were excluded because they had undergone surgery without a UGI pre-operative study due to an acute onset, 27/29 were surgically diagnosed with volvulus in IM and 2/29 with an apple peel intestinal atresia; of the 27 patients surgically diagnosed with volvulus in IM, 18/27 (66.7%) were younger than 1 year of life (range: 1 day–1 year of life, with a 55.5% of patients younger than 1 month of life), while 9/27 (33.3%) were older than 1 year of life (range 2 years–11 years). Further, 6 patients were excluded because UGI images were not available for review and 8 patients had already undergone surgery (jejunal atresia, duodenal stenosis, colic atresia, Hirschsprung disease) thus were non eligible for the study.

Twenty-nine out of 40 (72.5%) patients with surgically confirmed IM were younger than 2 years of life, 5/40 were aged 2–10 years, and 6 patients were older than 10 years of life (age range 0–15.8 years). Among patients with IM male: female ratio was 1:1.

As shown in Table 1, among patients younger than 2 years of life, 9 (31%) had an acute onset with abdominal distention and bilious vomiting, and 20 (69%) suffered from chronic digestive symptoms while among patients older than 2 years of life, 4 (36.36%) had an acute onset which required urgent surgery and the remaining 7 (63.63%) had a long story of recurrent vomits and frequent abdominal discomfort and/or failure to thrive. No statistical significant difference was detected (*p*-value = 1). Distribution of alternative diagnoses in those 15 patients non surgically confirmed with IM is shown in Table 2.

**Table 1:** Clinical presentation based on patient age

Age of onset	Acute Symptoms	Chronic Symptoms	Recurrent vomit + abdominal discomfort	Isolated nocturnal recurrent vomit	Recurrent vomit + failure to thrive
< 2y	9 (31%)	20 (69%)	12 (41%)	4 (13.8%)	4 (13.8%)
> 2y	4 (36.36%)	7 (63.63%)	3 (27.27%)	–	4 (36.36%)

**Table 2:** Alternative diagnoses of patients non surgically confirmed with IM

Diagnosis	N. of patients
Gastric emptying abnormalities	5
Neurological based gastric emptying abnormalities	5
Duodenal web	3
CIPO^2^	1
Intestinal subocclusion	1

1. Intestinal Malrotation; 2. Chronic Intestinal Pseudo-Obstruction

Overall the interobserver agreement on reporting UGI significant for malrotation was substantial (*k* = 0.75; 95% confidence interval CI 0.47–1.00) whereas the concordance with the surgical evidence of malrotation resulted fair (consultant radiologist_1_ *k* = 0.28; 95% CI 0.01–0.56; consultant radiologist_2_ *k* = 0.38; 95%CI 0.10; 0.66).

To further examine the discrepancy of these results, the inter-reader agreement was calculated for each UGI feature as reported in Table 3. The kappa values defined a barely fair agreement referring to the position of the third portion of the duodenum on lateral views, while a substantial agreement was achieved when defining the position of the DJJ on AP projections.

**Table 3:** Inter-radiologist concordance (Observer 1 vs Observer 2) for each UGI feature

	DJJ position	Duodenal lateral	Duodenal dilatation	Frist Jejunal loops	Caecum position
Kappa	0.70	0.23	0.54	0.89	0.50
(95% CI)	(0.49; 0.90)	(0.02; 0.4)	(0.27; 0.81)	(0.68; 1.00)	(0.13; 0.87)
(Observed Agreement)	(83.3%)	(52.9%)	(84.3%)	(93.5%)	(80.9%)

*UGI* upper Gastro-Intestinal, *DJJ* duodenal-jejunal junction, *CI* confidence interval

Diagnostic accuracy was then studied calculating Se, Sp, PPV, NPV for each feature evaluated by one radiologist and the association of two of them (position of DJJ and FJL).

As shown in Table 4, position of DJJ in AP projections resulted to be the most significant parameter demonstrating Se of 89% (95% CI 78.6–99.2) and Sp of 54% (95% CI 25.1–84), as well as a PPV of 86% and NPV of 60%.

**Table 4:** Diagnostic accuracy for each radiological parameter

	Se^6^	95% CI	Sp	95% CI	PPV	NPV
DJJ/surgery	0.89	0.78–0.99	0.54	0.25–0.84	0.86	0.6
Duodenum LL/surgery	0.8	0.65–0.94	0.33	0–0.71	0.85	0.25
DD/surgery	0.75	0.61–0.89	0.2	0–0.40	0.7	0.25
FJL/surgery	0.78	0.65–0.91	0.57	0.31–0.83	0.82	0.5
Caecum/surgery	0.88	0.74–1	0.16	0–0.46	0.76	0.33
DJJ + FJL/surgery	0.91	0.82–1	0.45	0.16–0.74	0.84	0.62
US-cD/surgery	0.63	0.41–0.84	1	1–1	1	0.3

*UGI* Upper Gastro-Intestinal series, *DJJ* duodenal-jejunal junction, *LL* latero-lateral projections, *DD* duodenal dilatation, *FJL* firsts jejunal loops, *US-cD* ultrasound-color Doppler, *Se* sensitivity, *SP* specificity, *PPV* positive predictive value, *NPV* negative predictive value

As shown in Table 5, in surgically confirmed malrotated patients distribution of UGI features of IM detected by each radiologist was calculated. The two radiologists had a broad agreement on all the features except for the altered position of the third portion of the duodenum on lateral views, therefore, it was not possible to describe the most occurring configuration in the population of this study.

**Table 5:** Distribution of UGI features in surgically confirmed malrotated patients of this study detected by each radiologist

	DJJ–4°quadrant	DJJ–L3 quadrant	Duodenal dilatation	FJL medialized/ right side of abdomen	Cecum altered position	Altered 3° portion of the duodenum, lateral view
Radiologist 1	67%	19%	24%	90%	70%	80%
Radiologist 2	70%	18%	22%	90%	70%	50%

*DJJ* duodenojejunal junction, *FJL* first jejunal loops

Us-Color Doppler study of the relative position of the mesenteric vessels was performed in 22 patients out of 55 of the study. The accuracy was calculated obtaining a Se of 0.63 (CI 95%: 0.41–0.84), a Sp of 1 (CI 95%: 1–1), with the PPV 1 and the NPV 0.30. Moreover, the accuracy analysis of UGI of these patients was then performed obtaining a Se of 0.84 (IC 95%: 0.67–1), a Sp of 0.66 (CI 85%: 0.13–1) with PPV 0.94 and NPV 0.40.

## Discussion

Isolated IM has been historically described as an infancy life-threatening condition, however the high prevalence of undiagnosed cases in older children and adults has been far demonstrated [19-21].

Although the classic representation of an infant affected by IM ranges from abdominal discomfort to possibly acute abdomen with bilious stained vomit, many recent studies suggest that the symptoms may depend on age at presentation, with atypical onset being more frequent among children older than 2 years of age [22, 23]. Thus, older patients may complain more often from recurrent epigastric pain or failure to thrive, intermittent vomiting, blood-stained stool, all symptoms assumed to result from intermitted, sub-occlusive volvulus [12, 24].

In line with literature data, the patients of this study with a surgically confirmed IM were mostly (72.5%) younger than 2 years of age with a significant rate (31%) of acute onset [22, 23, 25]. Patients older than 2 years of age had a similar occurrence of acute onset. This confirms the threat represented by acute midgut volvulus in malrotated patients which can occur at any age and must be considered when planning treatment in case of occasional diagnosis [26].

Undiagnosed IM is associated with a risk of Short Bowel Syndrome (SBS), even in case of minor intestinal surgical procedures. [27, 28] In addition to dependence on total parenteral nutrition, SBS may implicate many threatening complications such as cholestasis with liver failure, bowel dilatation, consequent bacterial overgrowth, and sepsis [29]. Currently the management of SBS is supportive, the surgical therapeutic options described in literature such as gut transplantation and autologous gut reconstruction are associated with a 10-years poor cumulative survival [30, 31].

Considering the clinical risks of unknown or misdiagnosed IM, the diagnosis must be as prompt and reliable as possible therefore ambiguous radiological elements need to be correctly understood [7-10, 19, 25, 31].

This study provides an analytical description of UGI focusing both on accepted and debatable features to properly value them in difficult cases.

The UGI is addressed as the preferred radiologic imaging procedure in suspected IM since the contrasted bolum progression allows the detection of duodenum position and possibly the presence of anomalous caliber variations [7, 16, 32, 33].

Anyway, referring to the pediatric population, many studies showed difficult UGI interpretation due to associated anatomical anomalies and procedure-related errors [10, 34]. In the attempt to objectivate the UGI study, Frederick et al. described four quadrants of the AP UGI scans useful in locating the DJJ [13]. Dekker et al. suggested the use of metal skin markers to reduce the interference of unintentional sideways rotation of the baby [35].

The grid presented in this study may help in making reproducible the reading of UGI by objectively locating many anatomical structures as the DJJ, the FJL and the caecum [13, 14] (Fig. 1).

The statistical analysis of the study showed a substantial agreement of imaging interpretation between two radiologists of different clinic background which clearly correlates with a shared imaging protocol. Despite the limited number of patients, some UGI parameters were significantly more associated with the surgical evidence of IM and more readable too. Images obtained with UGI AP projections were the most significant in terms of diagnosis. Specifically, DJJ and FJL position were the most represented and when considered together could reach a higher Se with a minimal expense on the Sp. On the other hand, based on Se and Sp, duodenal dilatation and caecum altered position demonstrated to be valuable as additional data when found positive in doubtful cases.

Even though UGI is classically described with the acquisition of lateral views, a barely fair agreement of the two radiologists was reached when describing these projections [17, 35]. The calculated Se and Sp of the lateral projections resulted valuable additional features in doubtful cases, though some practical consideration should be done. In fact, the low readability of this parameter may result from procedure-related errors in positioning the baby on one side at the time of acquisition of lateral views. Moreover, the lateral images lack in specific anatomical landmarks and the reading of the duodenal depiction may depend on the rotation of the baby [34]. Rotating the baby may impact on the quality of the AP images and jeopardize the depiction of the passage of the contrast medium through the DJJ, particularly in young children with a rapid passage of the contrasted bolus.

Analysis of pathological features frequency in patients with surgically confirmed IM showed that the most recurrent position of DJJ was in the fourth quadrant.

Aiming at improving the diagnostic process of suspected IM recent studies reported the use of Us-color Doppler with good diagnostic accuracy, supported by the absence of ionizing radiation exposure, non-invasiveness, rapidity, and repeatability [11, 36-38]. The diagnosis may depend on many aspects, such as superior mesenteric vessels relative position, or the orientation of the third portion of the duodenum which can be detected with the help of instillation of water in the stomach [38].

The retrospective review of preoperative US-color Doppler of this series showed a Sp of 1 which is a significant result. The comparative analyses of the US-color Doppler and UGI in these patients showed a good level of accuracy combining the two procedures.

Us-color Doppler may have some limits when unaccompanied by UGI, such as operator-dependency and presence of gas artifacts which reduce the quality of images and the accuracy and reliability of the exam [11, 39].

Results from this study show which of the features of the UGI are the most relevant in diagnosing IM and how to objectively detect them (the position of the DJJ and FJL, the presence of duodenal dilatations and eventually, with delayed scans, the position of the caecum). Moreover, the data analysis shows the weakness of the lateral projections which have no objective landmarks to be described by and thus risk to jeopardize IM diagnosis.

As a retrospective analysis, this study presents some limitations, especially the lack of complete data and a limited population. Selection bias may be present, as patients with acute volvulus accessing to the emergency room usually are sent directly to surgical evaluation without UGI.

## Conclusion

Based on this experience UGI on the sole AP projections seems to ensure a good diagnostic accuracy especially when associated with Us-color Doppler of the mesenteric vessels.

Further prospective and comparative studies are needed to better prove the reliability and reproducibility of a diagnostic protocol based on AP UGI and Us-color Doppler assessment and provide a standardized diagnosis method. Given the number of patients observed in our hospital over 14 years, a multicenter study may help to overcome the statistical power issue due to the low sample size of this study.

## Data Availability

The datasets generated during and/or analysed during the current study are available from the corresponding author on reasonable request.

## Abbreviations

AP: Antero-posterior
CIPO: Chronic Intestinal Pseudo-Obstruction
DJJ: Duodenal–jejunal junction
FJL: First jejunal loops
IM: Intestinal Malrotation
ISBS: Iatrogenic Short Bowel Syndrome
NPV: Negative predictive value
PPV: Positive predictive value
Se: Sensitivity
SMA: Superior mesenteric axis
Sp: Specificity
UGI: Upper gastro-intestinal series
